# Arterial Aging Best Reflected in Pulse Wave Velocity Measured from Neck to Lower Limbs: A Whole-Body Multichannel Bioimpedance Study

**DOI:** 10.3390/s22051910

**Published:** 2022-03-01

**Authors:** Ladislav Soukup, Pavel Jurak, Josef Halamek, Ivo Viscor, Magdalena Matejkova, Pavel Leinveber, Vlastimil Vondra

**Affiliations:** 1 The International Clinical Research Center, St. Anne’s University Hospital Brno, 656 91 Brno, Czech Republic; jurak@isibrno.cz (P.J.); josef@isibrno.cz (J.H.); magdalena.matejkova@fnusa.cz (M.M.); pavel.leinveber@fnusa.cz (P.L.); vond@isibrno.cz (V.V.); 2 The Institute of Scientific Instruments of the CAS, v.v.i, 612 64 Brno, Czech Republic; ivovi@isibrno.cz

**Keywords:** bioimpedance, pulse wave velocity (PWV), arterial stiffness

## Abstract

Pulse wave velocity is a commonly used parameter for evaluating arterial stiffness and the overall condition of the cardiovascular system. The main goal of this study was to establish a methodology to test and validate multichannel bioimpedance as a suitable method for whole-body evaluations of pulse waves. We set the proximal location over the left carotid artery and eight distal locations on both the upper and lower limbs. In this way, it was possible to simultaneously evaluate pulse wave velocity (PWV) in the upper and lower limbs and in the limbs via four extra PWV measurements. Data were acquired from a statistical group of 220 healthy subjects who were divided into three age groups. The data were then analysed. We found a significant dependency of aortic PWV on age in those values measured using the left carotid as the proximal. PWV values in the upper and lower limbs were found to have no significant dependency on age. In addition, the PWV in the left femoral artery shows comparable values to published already carotid-femoral values. Those findings prove the reliability of whole-body multichannel bioimpedance for pulse wave velocity evaluation and provide reference values for whole-body PWV measurement.

## 1. Introduction

The arterial system’s main function is to satisfy the needs of tissues and absorb pulsation blood flow from the left ventricle and convert it into a continuous flow. This enables continuous tissue oxygenation and lower heart strain. Greater arterial stiffness leads to an increase in pulse wave propagation, a decrease in blood volume accumulation during systole, an increase in systolic pressure, and a decrease in diastolic pressure. It gives rise to increased heart strain and lower tissue supply during diastole [1,2,3]. Because this is a physiological process, PWV should show an upward trend with aging [2,3,4].

Many techniques and parameters are used to evaluate arterial stiffness, including pulse wave analysis (PWA) [5,6,7], pulse wave intensity [8,9], and pulse wave velocity (PWV) [1,2,3,4,7]. Pulse wave velocity is a commonly used parameter in clinical practice. It refers to the velocity that the pulse wave reaches between proximal and distal locations and reflects to what extent the blood is decelerated by arterial compliance: the conversion of kinetic energy to potential energy. The PWV is defined as the ratio between the travelled distance (L_p-d_) and time delay (Δt) that the pulse wave needs to move from the proximal to the distal site:

(1) $$\mathrm{PWV}=\frac{L_{\text{p-d}}}{\Delta t}$$

The distance is commonly measured superficially between the proximal and distal sites [4,10,11]. Depending on which proximal site is used, certain subtraction methods or correction factors can be applied (e.g., the subject’s age). The subtraction method is used especially in the case of carotid–femoral pulse wave velocity with the applanation tonometry technique because the right carotid is used as the proximal site [3,11,12,13]. These distances are measured from the carotid artery to the suprasternal notch and from the femoral artery to the suprasternal notch. Finally, those are subtracted.

The situation surrounding time delay, or pulse transit time, is slightly more complicated. There are many techniques that can be used to acquire it, and each has some limitations. The most widely used approaches are applanation tonometry and Doppler ultrasound. Applanation tonometry allows for the measurement of the pressure wave only at those locations where the arteries are superficially palpated. This typically means the carotid, femoral, radial, or brachial arteries [7,11,14,15,16]. Doppler ultrasound has almost no limitations with respect to which locations can be used, but it requires the operator to have a high degree of skill. Both techniques largely acquire signals from the proximal and distal sites consequently. Because both signals require separate measurements, it is necessary to use some reference for time interval subtraction, commonly ECG. The proximal and distal time intervals are related to the R wave of ECG, and the final time delay is subtracted from the mean of a few consequent beats [10,11,14]. This can distort the final time delay and subsequently the PWV value due to changes in hemodynamic conditions.

As an alternative, it is possible to use other methods such as oscillometric [13,17,18], plethysmographic [19,20,21,22], and bioimpedance methods [4,23,24,25,26]. The bioimpedance method seems interesting especially because of its non-invasiveness, ease of use, the possibility to take measurements in several locations simultaneously, and its possibility of use during hemodynamic excitations. The original use of bioimpedance was to evaluate stroke volume and cardiac output. The pulsation bioimpedance component ΔZ corresponds to pulsation blood volume in the observed body part, or rather in the main arteries [27,28,29,30]. In most cases, one- or two-channel applications are used, whereas multichannel devices are largely associated with electrical impedance tomography (EIT) [31,32,33,34]. Only a few cardiological devices can measure three or four channels simultaneously [25,26]. The multichannel bioimpedance method enables the acquisition of bioimpedance in several channels simultaneously [23,24,30]. For evaluating PWV based on bioimpedance, it is necessary to determine the correct proximal channel, especially in cases of taking measurements from the heart. This is complicated because bioimpedance measures blood volume changes indirectly. In the case of the Multichannel Bioimpedance Monitor (MBM, ISI BRNO, Czech Republic), the proximal location is set based on a comparison of bioimpedance information with flow information using Doppler echocardiography as the channel located over the left carotid [23].

The main goal of this study was to introduce a methodology, test, and validate multichannel bioimpedance as a suitable method for whole-body evaluations of pulse wave velocity in a group of 200 volunteers. Moreover, reference values for whole-body PWV are presented.

## 2. Materials and Methods

The study was based on a group of 220 normal healthy subjects who had no history of disease with major impact on PWV values and took no related medications. Prior to the experiment, they completed a questionnaire about their health state and medication. The normal subjects were recruited from the general public based on their interest. The study group was composed of a similar proportion of men and women. The measurements were performed at a special laboratory with constant environmental conditions. For this study, a rest phase was used with spontaneous breathing lasting two minutes in the supine position on an examination bed while the subjects were awake.

The study was approved by the ethics committee at St. Anne’s University Hospital in Brno. All individual participants were fully informed and signed informed consent forms.

Data were acquired with a Multichannel Bioimpedance Monitor (MBM, Institute of Scientific Instruments of the Czech Academy of Sciences, Brno, Czech Republic) with up to 18 bioimpedance channels, a standard 12-lead ECG monitor (ECG12, Institute of Scientific Instruments of the Czech Academy of Sciences, Brno, Czech Republic), a Finometer Pro (Finapres Medical Systems B.V., Amsterdam, The Netherlands) for continuous blood pressure monitoring, and a phonocardiograph (PCG 1.0, Institute of Scientific Instruments of the Czech Academy of Sciences, Brno, Czech Republic) for heart sound recording. All signals were recorded with a sampling frequency of 500 Hz. In addition, blood pressure was acquired via a patient monitor (CARESCAPE™ B850, GE Healthcare, Chicago, IL, USA) at the beginning and the end of each measurement.

### 2.1. Multichannel Bioimpedance Measurement

**Figure 1:**
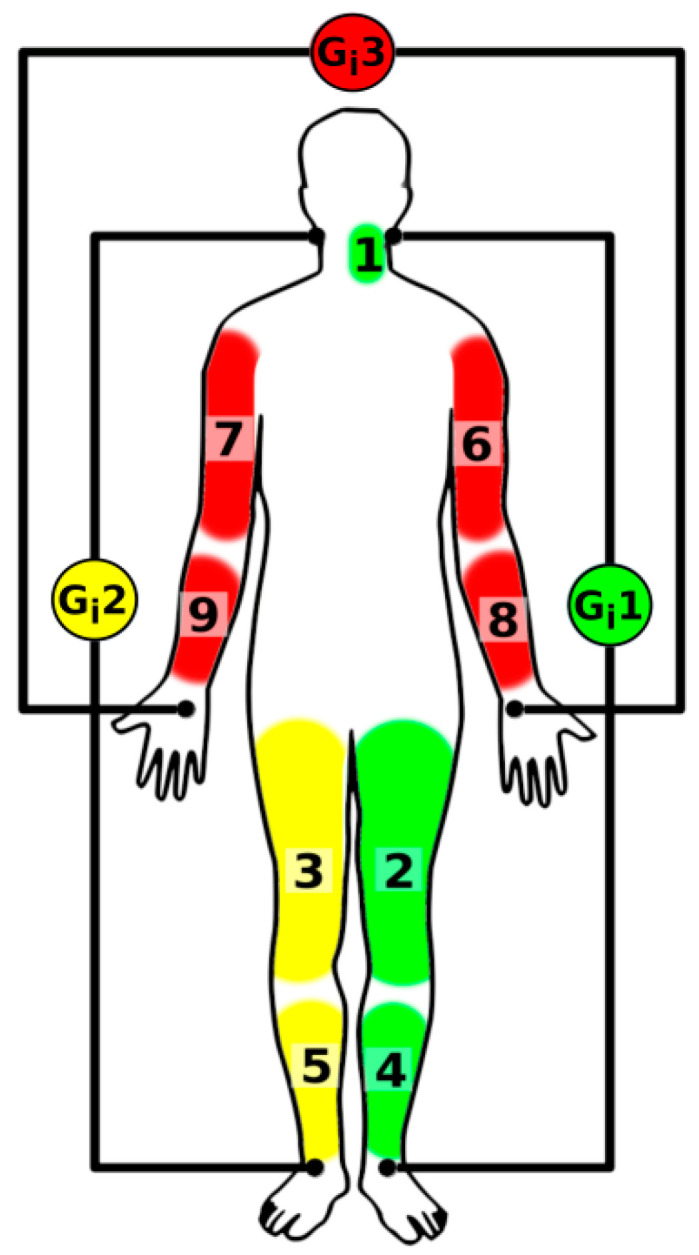
Localization of the bioimpedance channels (1–9) and current sources (G_i_1–G_i_3).

We focused on whole-body pulse wave velocity monitoring based on bioimpedance. Bioimpedance is measured indirectly using Ohm’s law; thus, the investigated body part is connected to an alternate current source and a voltage drop is observed. The study was based on the Multichannel Bioimpedance Monitor (MBM), which is able to measure blood flow properties in up to 18 channels using 3 current sources with different frequencies [24,30,35,36]. The default frequencies were set to 49 (G_i_1), 50 (G_i_2), and 51 kHz (G_i_3). The first current source (G_i_1) was placed between the left part of the neck and the left ankle, the second (G_i_2) was placed between the right neck and the right ankle, and the last (G_i_3) was placed between the left and right wrists, as shown in Figure 1 [24].

In this way, the bioimpedance channels placed on the left and right body parts were separated vertically, as well as the channels placed between hands horizontally. Individual bioimpedance channels were localized by their position and the frequency selectivity of the voltage detectors. Placement of the channels and matching current sources are shown in Figure 1 and Table 1.

The best proximal position was identified as the channel located over the left carotid (left neck) [23].

### 2.2. Pulse Wave Velocity Determination

Pulse wave velocity is defined as the ratio between distance and the corresponding time delay, as shown in Equation (1). The distance was measured directly from the sternum (at the level of second rib) to each proximal location, specifically to the center of the voltage electrode pairs. To determine the time delay, the pulsation component (ΔZ) of the bioimpedance signal was employed. Thus, for stroke volume, −dZ/dt max peak was chosen as the reference point to acquire the time delay. −dZ/dt max represents maximal axial blood acceleration [29]. The pulsation bioimpedance component ΔZ was acquired using bandpass filtration (Butterworth IIR filter; 0.6–18 Hz). Next, it was inverted, derived, and −dZ/dt max was detected for each channel [23,24,27,29,30].

**Figure 2:**
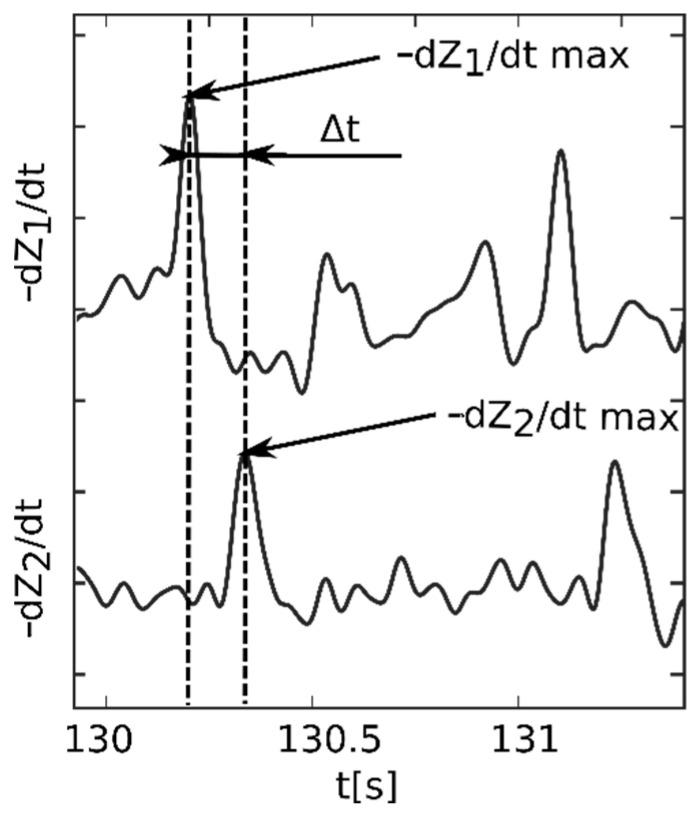
An example of a time delay (Δt) determination between a proximal location (left carotid −dZ_1_/dt) and a distal location (left thigh −dZ_2_/dt).

The time delay between proximal and distal locations, more precisely between the −dZ/dt max of those channels, could have thus been determined. An example of time delay determination is shown in Figure 2. The proximal reference location was set to the left carotid (channel 1) [23]. All of the eight remaining channels were used as distal locations. Moreover, to evaluate PWV in the upper and lower limbs, it was possible to use the channels closer to the heart as the proximal location (thighs and arms) and the distant locations (calves and forearms) as the distal location.

Finally, twelve pulse wave velocity values were acquired using Equation (1).

### 2.3. Statistical Analysis

The statistical analysis focused on the relationship between age and PWV.

First, the study group was divided into three subgroups based on age, namely 20–40, 40–60, and 60–80 years, and described by mean and standard deviations (Table 2). Next, representative PWV values were determined using mean and standard deviations for each age group and distal location. The next analysis focused on the effect of aging using regression analysis. For this, a linear polynomial was chosen:

(2) PWV=a×AGE+b

where *a* represents the slope of the line and *b* is a constant referred to as the y intercept. In order to simplify and make it more transparent, only two locations with the highest dependence of PWV on age were analyzed. The strongest dependence can be described by the slope of the line: parameter *a* in Equation (2). To select the best possible locations, the bootstrap function with one hundred repetitions was used. Thus, the polynomial equation was calculated one hundred times for a random sample of the studied group. After that, the mean value and standard deviation were computed based on the one hundred *a* constants of the polynomial equation. The two channels with the highest mean constants were selected for the next regression analysis as the representative.

In addition, a two-sided *t*-test was carried out to compare the difference between the age groups with the null hypothesis that two independent samples would have identical average values.

The data processing was performed using Python 5.

## 3. Results

Eight subjects had to be excluded because of the poor quality of their acquired signals. In addition, inappropriate subjects were excluded based on the well-known relationship between pulse wave velocity values and blood pressure [37,38,39]. The ESC/ESH Guidelines for managing arterial hypertension classify first hypertension grade with systolic pressure in the 150–159 mmHg range or diastolic pressure 90–99 mmHg [39]. Those subjects were included in the statistical analysis based on the finding that 72% of men and 65% of women in the Czech population aged 55–64 years have hypertension [40]. However, there were 22 subjects with systolic pressure in the 140–150 mmHg range and 3 subjects with diastolic pressure in the 90–96 mmHg range, mainly included in the age group of 60–80 years. Subjects with higher blood pressures were excluded.

The results are based on 200 normal healthy subjects with adequate signal quality. The subjects’ characteristics are presented in Table 2.

**Figure 3:**
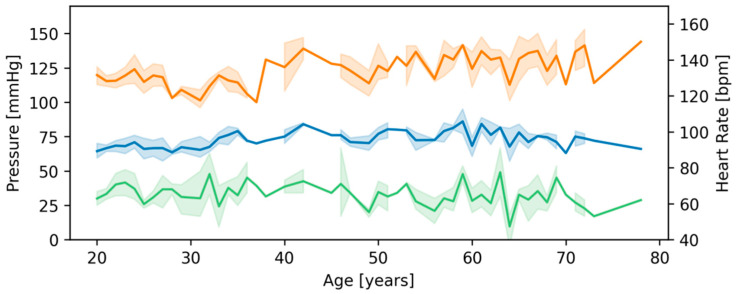
Lineplot representations, with 95% confidence intervals, of heart rate (green), systolic (orange) and diastolic (blue) pressures.

Hemodynamic parameters such as heart rate, systolic, and diastolic blood pressure for statistical groups are presented in Table 3 and Figure 3.

**Figure 4:**
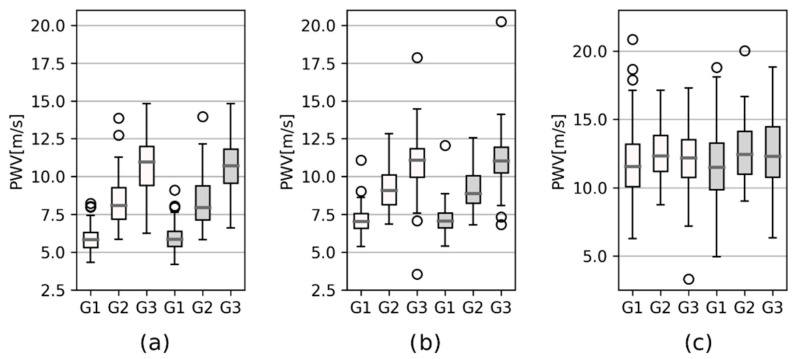
Boxplots of PWV from the left carotid (channel 1) to: (**a**) the thighs (channels 2 and 3); (**b**) the calves (channels 4 and 5); (**c**) between the thighs and calves, distinguished for left (white boxes) and right (grey boxes) body parts, and for age groups G1 (20–40 years), G2 (40–60 years), and G3 (60–80 years).

**Figure 5:**
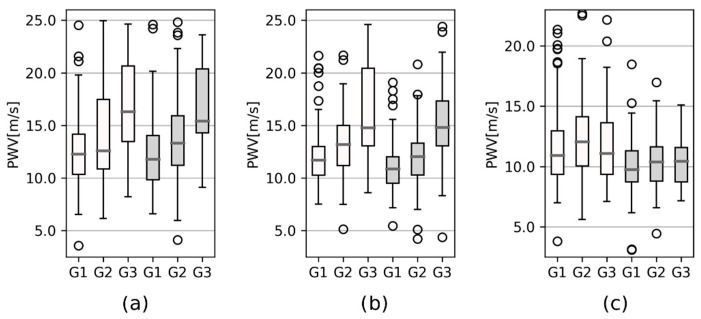
Boxplots of PWV from the left carotid (channel 1) to: (**a**) the arms (channels 6 and 7); (**b**) the forearms (channels 8 and 9); (**c**) from the arms to the forearms, distinguished for left (white boxes) and right (grey boxes) body part, and for age groups G1 (20–40 years), G2 (40–60 years), and G1 (60–80 years).

Representative pulse wave velocity values for the statistical group to the lower limbs are listed in Figure 4 and Table 4. Corresponding values for the upper limbs are shown in Figure 5 and Table 5.

Table 4 and Table 5 include reference PWV values, and regression parameter *a* represents the slope of the line described by Equation (2). Moreover, the *p*-value of the two-sided *t*-test compares the PWV values between the age groups.

**Figure 6:**
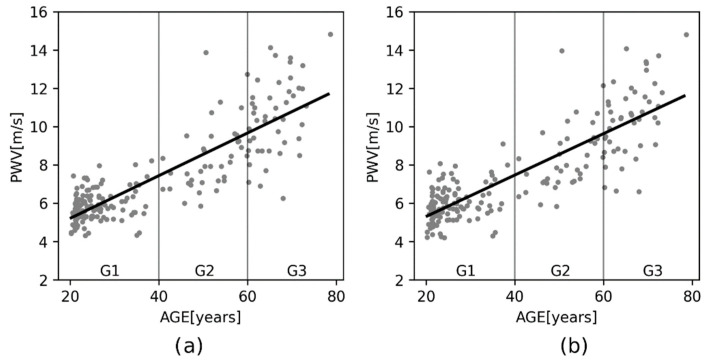
Plot of the regression analysis for: (**a**) the left thigh with a correlation coefficient of 0.842, where the black line is represented by the regression equation PWV = 0.111 × AGE + 3.021; (**b**) the right thigh with a correlation coefficient 0.839, where the black line is represented by the regression equation PWV = 0.108 × AGE + 3.154.

Based on the highest parameter *a* value from Table 4 and Table 5, channels 2 and 3 were chosen, corresponding with the left and right thighs as a representative example for performing the regression analysis. The relationship between pulse wave velocity and the subject’s age could be described by the regression equation PWV = 0.111 × AGE + 3.021 and a correlation coefficient 0.842 for the left thigh, and PWV = 0.108 × AGE + 3.154 and a correlation coefficient of 0.839 for the right thigh. These relationships are graphically represented in Figure 6.

## 4. Discussion

Based on previous findings, the bioimpedance method appears to be suitable for evaluating pulse wave velocity [4,23,25,26,27]. One proximal and one distal location are used in the case of commonly employed methods and bioimpedance. Nevertheless, proximal and distal locations are not measured simultaneously in most cases. This leads to a distortion of the PWV values due to changes in hemodynamic conditions such as heart rate and blood pressure. In addition, a high degree of professional experience is needed, especially for Doppler echocardiography and applanation tonometry. In comparison, the Multichannel Bioimpedance Monitor is a user-friendly device that enables the acquisition of blood volume change information in up to eighteen locations simultaneously [23,24,30]. However, methodology and reference values for whole-body (multichannel) applications were still lacking.

The distance measurement methodology was assumed from commonly used PWV measurement methods. Thus, it was measured directly on the body surface [1,4,6,15,27]. In case of time delay, it was necessary to correctly set the proximal and distal locations. The number of used locations for pulse wave velocity application was reduced to the nine most important: one proximal and eight distal. The selection of the correct proximal location was set to channel 1 over the left carotid based on a previous study [23].

The statistical analysis was based on the fact that arterial wall properties, such as elasticity, change with age; loss of elasticity results in an increase in PWV. This mainly appears in aortic PWV as the aorta is the most elastic human artery. The influence of aging on PWV values can be described by regression analysis. This is based on the highest slope of the regression line for channels 2 and 3, which were placed on the left and right thighs as being representative. The left thigh, or rather the left femoral artery, is commonly used for aortic PWV determination [2,3,4]. A strong dependence of PWV on aging described by the regression model for the right and left thigh was found. PWV dependency on age is mostly described by a polynomial of the second-order [4,41,42]. However, in this study, the second-order polynomial almost reached the parameters of a line, so the equation of a line seems to be better for making the description. Linear regression was used in [3], where the regression coefficient was 0.114 on a sample of 1087 subjects. Moreover, looking at Table 4 and Table 5, especially at the *p*-value, it is evident that pulse wave velocity changes with aging are more evident (*p* < 0.05) in those cases where the proximal location is set at the left carotid. Those segments include a number of large elastic arteries that lose compliance with aging. PWV in the upper and lower limbs showed no significant (0.081 < *p* < 0.900) changes with age in the case of normal healthy subjects.

It is well-known that arterial stiffness has an impact on blood pressure. Arterial elasticity loss leads to an increase in systolic pressure, a decrease in diastolic pressure, and a resulting increase in pulse pressure. However, an increase in pulse pressure is influenced by additional factors such as ventricular ejection, for example [43]. Diastolic pressure in addition to PWV can be marked as mechanical factors affecting cardiovascular risk [44]. A relationship between blood pressure and PWV can be described by the correlation coefficient. The PWV of the left thigh was chosen as representative because of its strongest dependence on age and its correspondence with carotid-femoral (aortic) PWV. We found that the correlation coefficient, with lower (rl) and upper (ru) bounds for a 95% confidence interval, was 0.397 (rl = 0.270, ru = 0.509) for diastolic, 0.534 (rl = 0.425, ru = 0.628) for systolic, and 0.320 (rl = 0.187, ru = 0.441) for pulse pressure for all subjects. Those findings show relationships that would be adequate for normal healthy subjects. The study was based on 174 subjects (59 ± 12.03 years old), where 47% had hypertension, and we obtains correlation coefficients of 0.110 for diastolic, 0.330 for systolic, and 0.363 for pulse pressure for all subjects [38]. All parameters were measured invasively along the descending thoracoabdominal aorta [38].

Comparing PWV values in the various age groups was more complicated. As was mentioned, the carotid-femoral PWV is considered the gold standard for evaluating central (aortic) pulse wave velocity. The pulse wave velocity at bioimpedance channel 2 (over the left femoral artery) has a similar predictive value as the carotid-femoral PWV. The values detected by bioimpedance were 5.86 ± 0.81 m/s for the 20–40 years group, 8.44 ± 1.68 m/s for those 40–60 years, and 10.79 ± 1.95 m/s for those 60–80 years. There are many studies with different statistical groups and different ways of dividing the subjects into groups [41,45,46,47]. In one study [41], carotid-femoral PWV was measured in 1455 subjects with optional or normal blood pressure and no cardiovascular risk factors. PWV reached values of 6.2 (4.7–7.6) m/s for the 30–39 years group, 7.2 (4.6–9.8) m/s for those 40–49 years, 8.3 (4.5–12.1) m/s for those 50–59 years, and 10.3 (5.5–15) m/s for those in the 60–69 years group. In some cases, brachial-ankle pulse wave velocity was used for a composite evaluation of central and peripheral arterial stiffness. However, there is no comparable equivalent in this study. Nevertheless, in another study [45] based on 79 subjects (75.7 ± 4.6 years), femoral-ankle PWV was acquired using applanation tonometry and an oscillometric device. The PWV values were obtained using a VP-1000 Plus waveform analyzer with a mean value of 10.84 ± 1.97 m/s, which is comparable to that of bioimpedance (from the left thigh to the left calf) 11.98 ± 2.54 m/s for the 60–80 years subgroup.

The absence of internal validation can be marked as a study limitation as well as the relatively small sample size. It would be more accurate to compare simultaneously measured bioimpedance and tonometry-based PWVs in the same subjects rather than performing a comparison with data from other studies.

## 5. Conclusions

Whole-body multichannel bioimpedance was used to take simultaneous measurements of pulse wave velocity. Based on an analysis, linear dependence exists between age and pulse wave velocity measured from the neck to the lower limbs for normal healthy subjects.

We suppose that a corresponding analysis of PWV may be used as a simple marker of arterial aging. Future perspectives include the acquisition and analysis of patients with specific diseases impacting PWV, such as hypertension and diabetes.

The whole-body multichannel bioimpedance allows for taking simultaneous measurements of proximal and distal sites to eliminate possible distortion. Moreover, it enables the evaluation of both central and peripheral PWV simultaneously without the need for highly skilled medical staff.

## Figures and Tables

**Table 1:** The bioimpedance channels (1–9) localization with corresponding current sources (G_i_1–3).

Bioimp. Channel	Current Source	Location
1	G_i_1	Neck left
2/3	G_i_1/G_i_2	Thigh left/right
4/5	G_i_1/G_i_2	Calf left/right
6/7	G_i_3	Arm left/right
8/9	G_i_3	Forearm left/right

**Table 2:** The statistical group’s characteristics.

		n	Age (Years)	Height (m)	Weight (kg)	BMI (kg/m^2^)
			Mean ± SD	Mean ± SD	Mean ± SD	Mean ± SD
G1	Women	56	26.3 ± 5.2	1.68 ± 0.07	63 ± 11	22.1 ± 3.5
	Men	62	24.7 ± 4.2	1.82 ± 0.07	80 ± 12	24.0 ± 3.1
	All	118	25.5 ± 4.7	1.76 ± 0.1	72 ± 14	23.1 ± 3.4
G2	Women	18	54.6 ± 5.4	1.66 ± 0.06	71 ± 13	26.1 ± 4.8
	Men	24	50.9 ± 5.5	1.8 ± 0.06	89 ± 13	27.6 ± 3.1
	All	42	52.5 ± 5.7	1.74 ± 0.1	82 ± 16	26.9 ± 4.0
G3	Women	17	68.2 ± 3.6	1.62 ± 0.09	77 ± 14	29.1 ± 4.4
	Men	23	66.2 ± 4.3	1.78 ± 0.06	89 ± 13	28.1 ± 3.7
	All	40	67.0 ± 4.1	1.71 ± 0.11	84 ± 15	28.6 ± 4.1

**Table 3:** Blood pressure and heart rate characteristic for the statistical group.

	Diastolic Pressure (mmHg)	Systolic Pressure (mmHg)	Pulse Pressure (mmHg)	Heart Rate (bpm)
	Mean ± SD	Mean ± SD	Mean ± SD	Mean ± SD
G1	68 ± 8	116 ± 12	48 ± 13	67 ± 10
G2	76 ± 7	127 ± 13	51 ± 10	65 ± 9
G3	75 ± 8	132 ± 14	57 ± 13	63 ± 10

**Table 4:** Reference PWV values (mean ± SD) to the lower limbs for age group G1 (20–40 years), G2 (40–60 years), G3 (60–80 years). Parameter *a* represents the mean ± SD of the slope of the regression line described by Equation (2); parameter *r* represents correlation coefficient.

Bioimp. Channel	*a* (-)	*r* (-)	PWV (m/s)	*p*	PWV (m/s)	*p*	PWV (m/s)
	Mean ± SD		G1	G1→G2	G2	G2→G3	G3
2	0.111 ± 0.006	0.842	5.86 ± 0.81	<0.05	8.44 ± 1.68	<0.05	10.79 ± 1.95
3	0.108 ± 0.005	0.839	5.93 ± 0.90	<0.05	8.40 ± 1.64	<0.05	10.73 ± 1.87
4	0.089 ± 0.006	0.786	7.10 ± 0.81	<0.05	9.30 ± 1.43	<0.05	10.89 ± 2.24
5	0.093 ± 0.007	0.806	7.12 ± 0.86	<0.05	9.25 ± 1.38	<0.05	11.23 ± 2.12
2_4	0.011 ± 0.010	0.091	1.82 ± 2.55	0.081	2.58 ± 1.87	0.233	11.98 ± 2.54
3_5	0.023 ± 0.009	0.181	1.66 ± 2.40	0.030	2.59 ± 2.23	0.743	12.42 ± 2.43

**Table 5:** Reference PWV values (mean ± SD) to the upper limbs for age group G1 (20–60 years), G2 (40–60 years), and G3 (60–80 years). Parameter *a* represents the mean ± SD of the slope of the regression line described by Equation (2); parameter *r* represents appropriate correlation coefficient.

Bioimp. Channel	*a* (-)	*r* (-)	PWV (m/s)	*p*	PWV (m/s)	*p*	PWV (m/s)
	Mean ± SD		G1	G1→G2	G2	G2→G3	G3
6	0.096 ± 0.018	0.390	12.58 ± 3.33	<0.05	14.23 ± 4.77	<0.05	16.83 ± 4.38
7	0.097 ± 0.016	0.397	12.34 ± 3.36	<0.05	14.18 ± 4.76	<0.05	16.42 ± 3.86
8	0.092 ± 0.015	0.454	12.00 ± 2.63	<0.05	13.47 ± 3.50	<0.05	16.27 ± 4.44
9	0.088 ± 0.013	0.481	11.00 ± 2.14	<0.05	12.06 ± 3.17	<0.05	15.52 ± 4.20
6_8	0.017 ± 0.014	0.087	11.60 ± 3.28	0.262	12.31 ± 3.78	0.795	12.09 ± 3.62
7_9	0.012 ± 0.008	0.095	9.96 ± 2.17	0.341	10.36 ± 2.41	0.900	10.42 ± 1.86

## Data Availability

Not applicable.

## References

[1] Salvi P. (2012). Pulse Waves: How Vascular Hemodynamics Affects Blood Pressure.

[2] Lehmann E.D. (1999). Clinical value of aortic pulse-wave velocity measurement. Lancet.

[3] Asmar R., Rudnichi A., Blacher J., London G.M., Safar M.E. (2001). Pulse pressure and aortic pulse wave are markers of cardiovascular risk in hypertensive population. Am. J. Hypertens..

[4] Koivistoinen T., Kööbi T., Jula A., Hutri-Kähönen N., Raitakari O.T., Majahalme S., Kukkonen-Harjula K., Lehtimäki T., Reunanen A., Viikari J. (2007). Pulse wave velocity reference values in healthy adults aged 26–75 years. Clin. Physiol. Funct. Imaging.

[5] Chowienczyk P. (2011). Pulse wave analysis: What do the numbers mean?. Hypertension.

[6] O’Rourke M.F., Jiang A.P.X.J. (2001). Pulse wave analysis. Br. J. Clin. Pharmacol..

[7] Gurovich A.N., Braith R.W. (2011). Pulse wave analysis and pulse wave velocity techniques: Are they ready for the clinic. Hypertens. Res..

[8] Alastruey J., Hunt A.A.E., Weinberg P.D. (2014). Novel wave intensity analysis of arterial pulse wave propagation accounting for peripheral reflections. Int. J. Numer. Methods Biomed. Eng..

[9] Sugawara M., Niki K., Ohte N., Okada T., Harada A. (2009). Clinical usefulness of wave intensity analysis. Med. Biol. Eng. Comput..

[10] Calabia J., Torguet P., Garcia M., Garcia I., Martin N., Guasch B., Faur D., Vallés M. (2011). Doppler ultrasound in the measurement of pulse wave velocity: Agreement with the complior method. Cardiovasc. Ultrasound.

[11] Jiang B., Liu B., McNeill K.L., Chowienczyk P.J. (2008). Measurement of pulse wave velocity using pulse wave Doppler ultrasound: Comparison with arterial tonometry. Ultrasound Med. Biol..

[12] Salvi P., Lio G., Labat C., Ricci E., Pannier B., Benetos A. (2004). Validation of a new non-invasive portable tonometer for determining arterial pressure wave and pulse wave velocity: The PulsePen device. J. Hypertens..

[13] Wassertheurer S., Kropf J., Weber T., van der Giet M., Baulmann J., Ammer M., Hametner B., Mayer C.C., Eber B., Magometschnigg D. (2010). A new oscillometric method for pulse wave analysis: Comparison with a common tonometric method. J. Hum. Hypertens..

[14] Vappou J., Luo J., Okajima K., Di Tullio M., Konofagou E. (2011). Aortic pulse wave velocity measured by pulse wave imaging (PWI): A comparison with applanation tonometry. Artery Res..

[15] Asmar R., Benetos A., Topouchian J., Laurent P., Pannier B., Brisac A.M., Target R., Levy B.I. (1995). Assessment of arterial distensibility by automatic pulse wave velocity measurement: Validation and clinical application studies. Hypertension.

[16] Salvi P., Grillo A., Parati G. (2015). Noninvasive estimation of central blood pressure and analysis of pulse waves by applanation tonometry. Hypertens. Res..

[17] Hametner B., Parragh S., Mayer C., Weber T., Van Bortel L., De Buyzere M., Segers P., Rietzschel E., Wassertheurer S. (2015). Assessment of model based (input) impedance, pulse wave velocity, and wave reflection in the asklepios cohort. PLoS ONE.

[18] Baulmann J., Schillings U., Rickert S., Uen S., Düsing R., Illyes M., Cziraki A., Nickering G., Mengden T. (2008). A new oscillometric method for assessment of arterial stiffness: Comparison with tonometric and piezo-electronic methods. J. Hypertens..

[19] Choi Y., Zhang Q., Ko S. (2013). Noninvasive cuffless blood pressure estimation using pulse transit time and Hilbert-Huang transform. Comput. Electr. Eng..

[20] Sone S., Hayase T., Funamoto K., Shirai A. (2017). Photoplethysmography and ultrasonic-measurement-integrated simulation to clarify the relation between two-dimensional unsteady blood flow field and forward and backward waves in a carotid artery. Med. Biol. Eng. Comput..

[21] Solà J., Chételat O., Sartori C., Allemann Y., Rimoldi S.F. (2011). Chest pulse-wave velocity: A novel approach to assess arterial stiffness. IEEE Trans. Biomed. Eng..

[22] Gomez-Clapers J., Casanella R., Pallas-Areny R. (2015). A novel method to obtain proximal plethysmographic information from distal measurements using the impedance plethysmogram. J. Electr. Bioimpedance.

[23] Soukup L., Hruskova J., Jurak P., Halamek J., Zavodna E., Viscor I., Matejkova M., Vondra V. (2019). Comparison of noninvasive pulse transit time determined from Doppler aortic flow and multichannel bioimpedance plethysmography. Med. Biol. Eng. Comput..

[24] Vondra V., Jurak P., Viscor I., Halamek J., Leinveber P., Matejkova M., Soukup L. (2016). A multichannel bioimpedance monitor for full-body blood flow monitoring. Biomed. Tech..

[25] Kööbi T., Kähönen M., Iivainen T., Turjanmaa V. (2003). Simultaneous non-invasive assessment of arterial stiffness and haemodynamics—A validation study. Clin. Physiol. Funct. Imaging.

[26] Kusche R., Klimach P., Ryschka M. (2018). A multichannel real-time bioimpedance measurement device for pulse wave analysis. IEEE Trans. Biomed. Circuits Syst..

[27] Soukup L., Vondra V., Viscor I., Jurak P., Halamek J. Pulse wave velocity and cardiac output vs. heart rate in patients with an implanted pacemaker based on electric impedance method measurement. Proceedings of the XV International Conference on Electrical Bio-Impedance (ICEBI) & XIV Conference on Electrical Impedance Tomography (EIT).

[28] Bernstein D.P. (2010). Impedance cardiography: Pulsatile blood flow and the biophysical and electrodynamic basis for the stroke volume equations. J. Electr. Bioimpedance.

[29] Bernstein D.P., Lemmens H.J.M. (2005). Stroke volume equation for impedance cardiography. Med. Biol. Eng. Comput..

[30] Langer P., Jurák P., Vondra V., Halámek J., Mešťaník M., Tonhajzerová I., Viščor I., Soukup L., Matejkova M., Závodná E. (2018). Respiratory-induced hemodynamic changes measured by whole-body multichannel impedance plethysmography. Physiol. Res..

[31] Halter R.J., Hartov A., Paulsen K.D. (2008). A broadband high-frequency electrical impedance tomography system for breast imaging. IEEE Trans. Biomed. Eng..

[32] Otten D.M., Rubinsky B. (2000). Cryosurgical monitoring using bioimpedance measurements—A feasibility study for electrical impedance tomography. IEEE Trans. Biomed. Eng..

[33] Paulson K., Lionheart W., Pidcock M. (1993). Optimal experiments in electrical impedance tomography. IEEE Trans. Med. Imaging.

[34] Saulnier G.J., Blue R.S., Newell J.C., Isaacson D., Edic P.M. (2001). Electrical impedance tomography. IEEE Signal Process. Mag..

[35] Matejkova M., Vondra V., Soukup L., Plesinger F., Viscor I., Halamek J., Jurak P. (2015). Changes of pulse wave velocity in the lower limbs in hypertensive patients. Proceedings of the Computing in Cardiology.

[36] Vondra V., Jurak P., Halamek J., Viscor I. (2015). Device for Blood Flow Property Measurement and Method of Its Connection. U.S. Patent.

[37] Koivistoinen T., Lyytikäinen L.P., Aatola H., Luukkaala T., Juonala M., Viikari J., Lehtimäki T., Raitakari O.T., Kähönen M., Hutri-Kähönen N. (2018). Pulse wave velocity predicts the progression of blood pressure and development of hypertension in young adults. Hypertension.

[38] Kim E.J., Park C.G., Park J.S., Suh S.Y., Choi C.U., Kim J.W., Kim S.H., Lim H.E., Rha S.W., Seo H.S. (2006). Relationship between blood pressure parameters and pulse wave velocity in normotensive and hypertensive subjects: Invasive study. J. Hum. Hypertens..

[39] Williams B., Mancia G., Spiering W., Rosei E.A., Azizi M., Burnier M., Clement D.L., Coca A., De Simone G., Dominiczak A. (2018). 2018 ESC/ESH guidelines for themanagement of arterial hypertension. Eur. Heart J..

[40] Widimsky J., Filipovsky J., Ceral J., Cifkova R., Linhart A., Monhart V., Rosolova H., Seidlerova Mlikova J., Soucek M., Spinar J. (2018). Diagnosticke a lecebne postupy u arterialni hypertenze—Verze 2017. Doporuceni ceske spolecnosti pro hypertenzi. Vnitr Lek.

[41] Mattace-Raso F.U.S., Hofman A., Verwoert G.C., Wittemana J.C.M., Wilkinson I., Cockcroft J., McEniery C., Yasmina, Laurent S., Boutouyrie P. (2010). Determinants of pulse wave velocity in healthy people and in the presence of cardiovascular risk factors: ‘Establishing normal and reference values’. Eur. Heart J..

[42] McEniery C.M., Yasmin, Hall I.R., Qasem A., Wilkinson I.B., Cockcroft J.R. (2005). Normal vascular aging: Differential effects on wave reflection and aortic pulse wave velocity: The anglo-cardiff collaborative trial (ACCT). J. Am. Coll. Cardiol..

[43] Nichols W.W., O’Rourke M.F., Vlachopoulos C., Hoeks A.P., Reneman R.S. (2011). McDonald’s Blood Flow in Arteries Theoretical, Experimental and Clinical Principles.

[44] Blacher J., Asmar R., Djane S., London G.M., Safar M.E. (1999). Aortic pulse wave velocity as a marker of cardiovascular risk in hypertensive patients. Hypertension.

[45] Meyer M.L., Tanaka H., Palta P., Patel M.D., Camplain R., Couper D., Cheng S., Al Qunaibet A., Poon A.K., Heiss G. (2016). Repeatability of central and peripheral pulse wave velocity measures: The atherosclerosis risk in communities (ARIC) study. Am. J. Hypertens..

[46] Baier D., Teren A., Wirkner K., Loeffler M., Scholz M. (1234). Parameters of pulse wave velocity: Determinants and reference values assessed in the population-based study LIFE-adult. Clin. Res. Cardiol..

[47] Diaz A., Zócalo Y., Bia D., Wray S., Fischer E.C. (2018). Reference intervals and percentiles for carotid-femoral pulse wave velocity in a healthy population aged between 9 and 87 years. J. Clin. Hypertens..

